# Contrast enhanced cardiovascular magnetic resonance imaging prior to prophylactic implantation of a cardioverter/defibrillator identifies patients with increased risk for ventricular arrhythmias

**DOI:** 10.1186/1532-429X-11-S1-O1

**Published:** 2009-01-28

**Authors:** Philipp Boyé, Hassan Abdel-Aty, Udo Zacharzowsky, Alexander Schirdewan, Rainer Dietz, Jeanette Schulz-Menger

**Affiliations:** Franz-Volhard Clinic, Charite Campus Buch, Berlin, Germany

**Keywords:** Ventricular Arrhythmia, Late Gadolinium Enhancement, Cardiovascular Magnetic Resonance Imaging, Remote Myocardium, Chronic Myocardial Infarction

## Introduction

Prophylactic implantation of a cardioverter/defibrillator (ICD) has been shown to reduce mortality in patients with chronic myocardial infarction (CMI) and an increased risk for life threatening ventricular arrhythmia (VA). The use of ICDs in this large patient population is still limited by high costs and possible adverse events including inappropriate discharges and progression of heart failure. VA is related to infarct size and seems to be related to infarct morphology. Contrast enhanced cardiovascular magnetic resonance imaging (ceCMR) can detect and quantify myocardial fibrosis in the setting of CMI and might therefore be a valuable tool for a more accurate risk stratification in this setting.

## Hypothesis

ceCMR can identify the subgroup developing VA in patients with prophylactic ICD implantation following MADIT criteria.

## Methods

We prospectively enrolled 52 patients (49 males, age 69 ± 10 years) with CMI and clinical indication for ICD therapy following MADIT criteria. Prior to implantation (36 ± 78 days) patients were investigated on a 1.5 T clinical scanner (Siemens Avanto^©^, Germany) to assess left ventricular function (LVEF), LV end-diastolic volume (LVEDV) and LV mass (sequence parameters: GRE SSFP, matrix 256 × 192, short axis stack; full LV coverage, no gap; slice thickness 6 mm). For quantitative assessment of infarct morphology late gadolinium enhancement (LGE) was performed including measurement of total and relative infarct mass (related to LV mass) and the degree of transmurality (DT) as defined by the percentage of transmurality in each scar. (sequence parameters: inversion recovery gradient echo; matrix 256 × 148, imaging 10 min after 0.2 μg/kg gadolinium DTPA; slice orientation equal to SSFP). MRI images were analysed using dedicated software (MASS^©^, Medis, Netherlands). LGE was defined as myocardial areas with signal intensity above the average plus 5 SD of the remote myocardium. After implantation, patients were followed up including ICD readout after 3 and than every 6 months for a mean of 945 ± 344 days. ICD data were evaluated by an experienced electrophysiologist. Primary endpoint was the occurrence of an appropriate discharge (DC), antitachycard pacing (ATP) or death from cardiac cause.

## Results

The endpoint occurred in 10 patients (3 DC, 6 ATP, 1 death). These patients had a higher relative infarct mass (28 ± 7% vs. 22 ± 11%, p = 0.03) as well as high degree of transmurality (64 ± 22% vs. 44 ± 25%, p = 0.05). Their LVEF (29 ± 8% vs. 30 ± 4%, p = 0.75), LV mass (148 ± 29 g vs. 154 ± 42 g, p = 0.60), LVEDV (270 ± 133 ml vs. 275 ± 83 ml, p = 0.90) or total infarct mass (43 ± 19 g vs. 37 ± 21 g, p = 0.43) were however not significant from the group with no events. In a cox proportional hazards regression model including LVEF, LVEDV, LV mass, DT and age, only degree of transmurality and relative infarct mass emerged as independent predictors of the primary end point (p = 0.009)

## Conclusion

In CMI-patients fulfilling MADIT criteria ceCMR could show that the extent and transmurality of myocardial scarring are independent predictors for life threatening ventricular arrhythmia or death. This additional information could lead to more precise risk stratification and might reduce adverse events and cost of ICD therapy in this patient population. Larger trials are needed to confirm this finding.

